# Saponins from *Allium macrostemon Bulbs* Attenuate Endothelial Inflammation and Acute Lung Injury via the NF-κB/VCAM-1 Pathway

**DOI:** 10.3390/molecules29061239

**Published:** 2024-03-11

**Authors:** Li Liu, Liang Qiu, Jing Xue, Chao Zhong, Manman Qin, Yifeng Zhang, Chuanming Xu, Yanfei Xie, Jun Yu

**Affiliations:** 1 Center for Translational Medicine, Jiangxi University of Chinese Medicine, Nanchang 330004, China; 2 Department of Cardiovascular Sciences and Center for Metabolic Disease Research, Lewis Katz School of Medicine, Temple University, Philadelphia, PA 19140, USA

**Keywords:** saponins from *Allium macrostemon bulbs*, endothelial inflammation, VCAM-1, acute lung injury

## Abstract

Endothelial inflammation is a multifaceted physiological process that plays a pivotal role in the pathogenesis and progression of diverse diseases, encompassing but not limited to acute lung infections like COVID-19, coronary artery disease, stroke, sepsis, metabolic syndrome, certain malignancies, and even psychiatric disorders such as depression. This inflammatory response is characterized by augmented expression of adhesion molecules and secretion of pro-inflammatory cytokines. In this study, we discovered that saponins from *Allium macrostemon bulbs* (SAMB) effectively inhibited inflammation in human umbilical vein endothelial cells induced by the exogenous inflammatory mediator lipopolysaccharide or the endogenous inflammatory mediator tumor necrosis factor-α, as evidenced by a significant reduction in the expression of pro-inflammatory factors and vascular cell adhesion molecule-1 (VCAM-1) with decreased monocyte adhesion. By employing the NF-κB inhibitor BAY-117082, we demonstrated that the inhibitory effect of SAMB on VCAM-1 expression may be attributed to the NF-κB pathway’s inactivation, as characterized by the suppressed IκBα degradation and NF-κB p65 phosphorylation. Subsequently, we employed a murine model of lipopolysaccharide-induced septic acute lung injury to substantiate the potential of SAMB in ameliorating endothelial inflammation and acute lung injury in vivo. These findings provide novel insight into potential preventive and therapeutic strategies for the clinical management of diseases associated with endothelial inflammation.

## 1. Introduction

Endothelial cells (ECs) line the inner walls of all blood vessels and are critical in maintaining hemostasis, regulating vascular tone and endothelial permeability, and suppressing leukocyte activation [1,2,3]. Resting ECs do not engage in interactions with leukocytes [4]. This is due to their sequestration of leukocyte-interactive proteins, such as P-selectin and chemokines, within specialized secretory vesicles called Weibel–Palade bodies (WPBs) [5,6]. Additionally, resting ECs maintain low transcription of other adhesion molecules, including E-selectin, VCAM-1, and intercellular adhesion molecule-1 (ICAM-1) [7]. The failure of ECs to adequately perform these basal functions is referred to as “EC dysfunction”, which can contribute to the development of various diseases, including but not limited to acute lung infections like COVID-19 [8], coronary artery disease [9], stroke [10], sepsis [11], metabolic syndrome [12], certain malignancies [13], and even psychiatric disorders such as depression [14]. The role of inflammation in EC dysfunction is well established [15], and ICAM-1 is widely recognized as a reliable marker indicating EC infiltration and activation [16,17]. Considering the numerous side effects and drug resistance associated with anti-inflammatory therapies like ketoconazole, activated protein C, and glucocorticoids [18,19], there is an unmet clinical need to develop safer and more effective medications for inflammation.

The pulmonary endothelium plays a crucial role in the pathogenesis of acute lung injury (ALI), as it serves as a primary target for injury-related circulating cells and humoral mediators [20]. The interaction between leukocytes and ECs constitutes a pivotal step in the progression of ALI. Leukocytes’ adhesion to and migration across ECs are facilitated by reciprocal interactions between complementary adhesion molecules expressed in both cell types. The increased expression or release of endothelial cell adhesion molecules is a hallmark of endothelial cell activation [21]. Upon the infiltration of leukocytes into the pulmonary parenchyma, they can release inflammatory mediators to eliminate pathogens; however, excessive immune activation has the potential to disrupt the delicate balance between pro-inflammatory and anti-inflammatory mechanisms, thereby triggering a “cytokine storm” and subsequent tissue damage [22].

*Allii macrostemonis bulbs* (AMBs), renowned in traditional Chinese medicine, have been highly valued for their medicinal and dietary properties since ancient times [23]. AMBs and their prescriptions exhibit various activities, including anti-platelet aggregation [24], lipid-lowering [25], antitumor [26], antibacterial [27], and antidepressant effects [28], along with antioxidant potential [29] and analgesic activity [30]. Furthermore, previous studies have demonstrated the potential of AMBs in protecting endothelial function; however, these studies were primarily focused on AMBs’ antioxidant properties and regulation of nitric oxide production [31,32,33]. There is still a lack of investigations on AMBs’ regulation of endothelial function. The current study aimed to understand how SAMB (saponins from AMBs), the main active component of AMBs [34], protect ECs by inhibiting inflammation in vitro and in vivo. In vitro, we utilized both the exogenous inflammatory mediator lipopolysaccharide (LPS) and the endogenous inflammatory mediator tumor necrosis factor-α (TNF-a) to investigate the impact of SAMB on inflammation in human umbilical vein ECs (HUVECs). In vivo, we induced ALI in C57BL/6 mice by intraperitoneal administration of LPS to determine the therapeutic potential of SAMB for septic ALI, potentially offering novel preventive and therapeutic approaches for the clinical management of ALI.

## 2. Results

### 2.1. SAMB Did Not Affect the Proliferation and Migration of ECs

**Figure 1:**
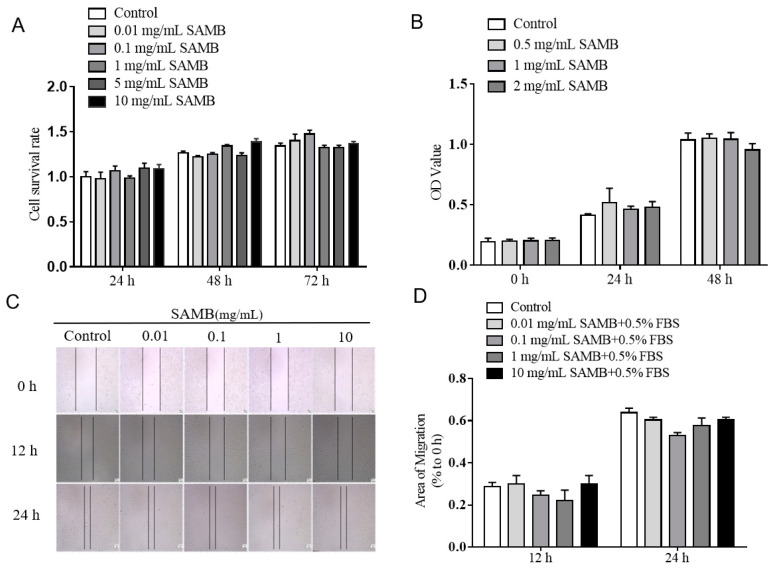
The effects of SAMB on the proliferation and migration of HUVECs: (**A**) CCK8 assays for cell viability. (**B**) CCK8 assays for cell proliferation. (**C**) Representative pictures of cell migration (×50). (**D**) Analysis of migration area.

As shown in Figure 1A, the Cell Counting Kit-8 (CCK-8) assay demonstrated that SAMB (0.01, 0.1, 1, 5, and 10 mg/mL) did not exhibit significant cytotoxicity towards HUVECs, as well as measuring the cell proliferation and migration rates of HUVECs (Figure 1B–D). Endothelial proliferation and migration are pivotal processes involved in inflammation [35]. Thus, these findings indicate that SAMB does not influence inflammation by affecting the basic functions of ECs.

### 2.2. SAMB Inhibited LPS-Induced Inflammation in HUVECs

**Figure 2:**
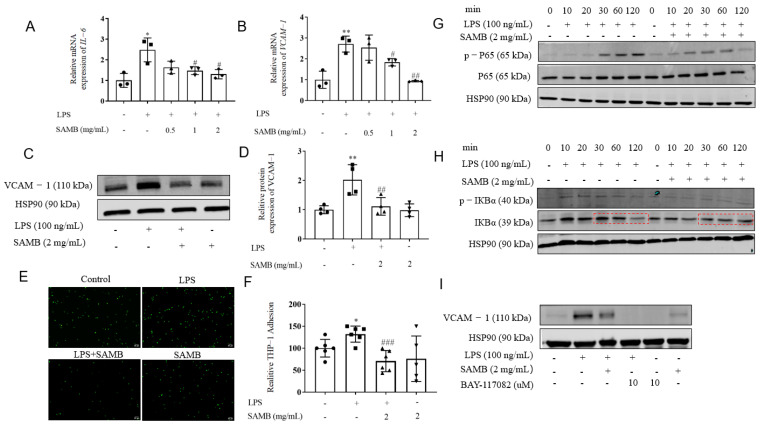
SAMB inhibited inflammation and monocyte adhesion induced by LPS in HUVECs: (**A**–**C**) HUVECs were pretreated with different concentrations of SAMB (0.5, 1, 2 mg/mL) for 24 h and then stimulated with LPS (100 ng/mL) for 6 h (n = 3). The mRNA expression of (**A**) *Vcam-1* and (**B**) *Il-6*; (**C**) Western blotting of VCAM-1 in HUVECs; (**D**) quantitation analysis of protein expression of VCAM-1 (n = 4); (**E**,**F**) representative images and quantification of the adhesion of THP-1 to HUVECs observed under a fluorescence microscope (×100), n = 5–6; (**G**) Western blotting of p-p65 and p65 in HUVECs; (**H**) Western blotting of p-IκBα and IκBα in HUVECs; (**I**) the protein expression of VCAM-1 caused by stimulating HUVECs with BAY-117082 for 1 h and then 100 ng/mL LPS for 24 h. Data are shown as the mean ± SD; * *p* < 0.05, ** *p* < 0.01 vs. control group; # *p* < 0.05, ## *p* < 0.01, ### *p* < 0.001 vs. LPS+SAMB group.

To examine the anti-inflammatory effect of SAMB, HUVECs were pretreated with various doses of SAMB or vehicle and subjected to LPS stimulation to elicit endothelial inflammation. The expression of inflammatory factors and adhesion molecules was accessed by RT-qPCR. The results revealed a significant upregulation of *Il-6* and *Vcam-1* mRNA expression in response to LPS stimulation, which was dose-dependently attenuated by SAMB (Figure 2A,B). The Western blot analysis revealed a significant decrease in the protein expression of VCAM-1 following SAMB treatment (Figure 2C,D), thereby leading to a reduction in the firm adhesion of calcein-AM-labeled THP-1 cells to LPS-activated HUVECs (Figure 2E,F). However, no significant alterations were observed in the mRNA and protein expression of ICAM-1 in response to SAMB treatment (Figure S1).

To further elucidate the underlying molecular mechanisms, we assessed the expression of proteins associated with the NF-κB pathway. The findings revealed that SAMB treatment significantly inhibited the phosphorylation of NF-κB p65 induced by LPS (Figure 2G). IκBα, an inhibitory molecule of NF-κB, undergoes a series of post-translational modifications upon LPS stimulation: IκBα is first phosphorylated by IKK and subsequently ubiquitinated by β-TrCP, leading to recognition and degradation by the 26S proteasome, ultimately resulting in the release and activation of NF-κB from the cytoplasm [36]. Upon LPS stimulation, we observed no alteration in the protein levels of phosphorylated and total IKK in HUVECs when co-cultured with SAMB (Figure S2A). Meanwhile, the expression levels of IκBα were significantly reduced at 120 min following LPS induction, which was attenuated by SAMB, implying the inhibition of the NF-κB pathway (Figure 2H). The MAPK pathway was also extensively implicated in the inflammatory response [37]. However, SAMB did not exert any discernible effects on the phosphorylation of JNK and ERK (Figure S2B–D).

It has been extensively reported that activation of the NF-κB pathway can induce the upregulation of the adhesion molecules ICAM-1 and VCAM-1 in HUVECs [38]. To determine whether the effect of SAMB on VCAM-1 expression is NF-κB-dependent, we pretreated HUVECs with BAY11-7082 (10 μM), a selective and irreversible inhibitor of the NF-κB pathway, for 1 h before LPS stimulation on SAMB- or vehicle-pretreated HUVECs. The findings revealed that BAY11-7082 displayed a robust inhibitory effect on the LPS-induced expression of VCAM-1 in vehicle-treated cells, while the downregulation of VCAM-1 mediated by SAMB may be dependent on NF-κB inhibition, which warrants further investigation (Figure 2I).

### 2.3. SAMB Inhibited TNF-α-Induced Inflammation in HUVECs

**Figure 3:**
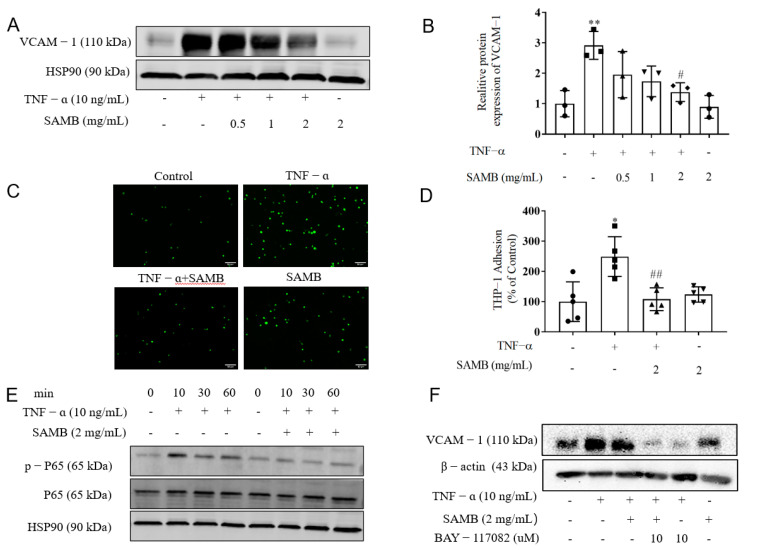
SAMB inhibited VCAM-1 expression and monocyte adhesion induced by TNF-α in HUVECs: (**A**) Western blotting of VCAM-1 in HUVECs after their stimulation with TNF-α (10 ng/mL). (**B**) Quantitation analysis of protein expression of VCAM-1 (n = 3). (**C**,**D**) Representative images and quantification of the adhesion of THP-1 cells to HUVECs, observed under a fluorescence microscope. (×200). (**E**) Western blotting of p-p65 and p65 in HUVECs. (**F**) The relative quantitation of the protein levels in HUVECs. (**F**) The protein expression of VCAM-1 in HUVECs stimulated with BAY-117082 for 1 h and then 10 ng/mL TNF-α for 24 h. Data are shown as the mean ± SD; * *p* < 0.05, ** *p* < 0.01 vs. control group; # *p* < 0.05, ## *p* < 0.01 vs. LPS+SAMB group.

To examine whether SAMB attenuates non-pathogen-associated molecular pattern (PAMP)-induced inflammation, we used a TNF-α-induced EC activation model. The pro-inflammatory cytokine TNF-α represents a prototypical endogenous factor synthesized by macrophages and other immune cells, actively participating in the pathogenesis of numerous chronic inflammatory diseases [39]. In the HUVEC inflammation model induced by TNF-α, we further substantiated the anti-inflammatory efficacy of SAMB, demonstrating outcomes analogous to those observed in the LPS-induced HUVEC inflammation model. As shown in Figure 3A,B, SAMB dose-dependently attenuated TNF-α-induced VCAM-1 expression, thereby leading to a reduction in the firm adhesion of THP-1 cells to TNF-α-activated HUVECs (Figure 3C,D). Furthermore, the phosphorylation of NF-κB p65 induced by TNF-α was significantly inhibited by SAMB treatment (Figure 3E). Subsequently, we observed a significant inhibition of TNF-α-induced VCAM-1 expression by BAY-117082; however, the combination with SAMB did not result in an additive inhibitory effect. This finding suggests that the inhibitory effect of SAMB on VCAM-1 expression is mediated through the inactivation of the NF-κB pathway (Figure 3F). In conclusion, SAMB exerted a downregulatory effect on the expression of VCAM-1 by inhibiting the NF-κB pathway, thereby reducing the TNF-α-induced adherence of monocytes to HUVECs.

### 2.4. SAMB Mitigated Acute Pulmonary Tissue Damage and Suppressed the Inflammatory Response in LPS-Induced ALI

**Figure 4:**
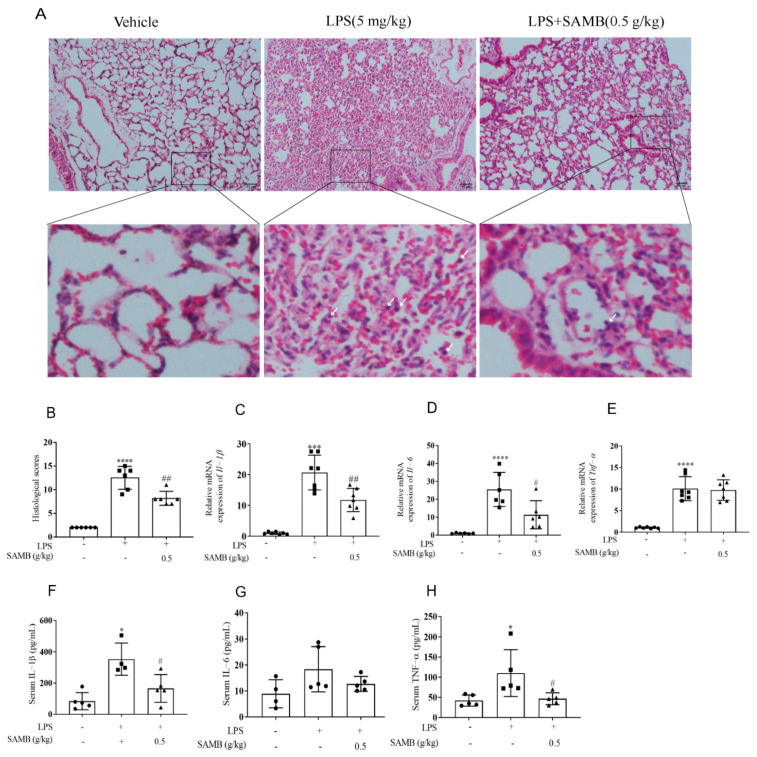
SAMB alleviated acute lung injury in C57BL/6 mice. The C57BL/6 mice were orally administered with 0.5 g/kg SAMB for a duration of 7 days, followed by an intraperitoneal injection of LPS at a dose of 5 mg/kg. Lung tissue was collected after 24 h. (**A**) Representative images of the histological damage in lungs after the LPS challenge, examined by HE staining (×100); the white arrows in this partial zoom indicate the presence of inflammatory cells. (**B**) Histological score of lung tissue (n = 6). The relative expression of (**C**) *Il-1β*, (**D**) *Il-6*, and (**E**) *Tnf-α* in the lungs was measured by real-time PCR (n = 7). The contents of (**F**) IL-1β, (**G**) IL-6, and (**H**) TNF-α in the serum were detected by ELISA (n = 4–5). Data are shown as the mean ± SD; * *p* < 0.05, *** *p* < 0.001, **** *p* < 0.0001 vs. control group; # *p* < 0.05, ## *p* < 0.01, vs. LPS+SAMB group.

Septic ALI was induced in C57BL/6 mice by intraperitoneal administration of LPS, as described in the previous literature [40]. The pathological alterations were assessed through H&E staining, enabling the evaluation of lung tissue damage based on the extent of inflammatory cell infiltration, edema, and interstitial lung thickness [41]. The model group exhibited significant lung damage, as characterized by neutrophil infiltration and thickening of the alveolar wall. SAMB (0.5 g/kg) significantly improved the histological damage caused by LPS (Figure 4A,B). Subsequently, we conducted further investigations into the alterations in inflammatory factors within lung tissues and sera. The mRNA levels of *Il-1β*, *Tnf-α*, and *Il-6* exhibited a significant increase in the LPS-treated mice. The administration of SAMB resulted in a reduction in *Il-6* and *Il-1β* mRNA. Moreover, SAMB significantly attenuated the LPS-induced elevation of serum levels of IL-1β and TNF-α (Figure 4F–H). These findings suggest that SAMB effectively ameliorated ALI and systemic inflammation during the LPS challenge.

### 2.5. SAMB Reduced VCAM-1 Expression in the Pulmonary Tissue of ALI Mice

**Figure 5:**
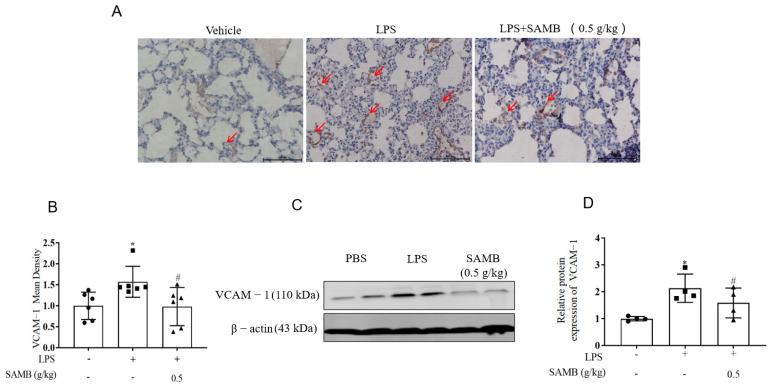
Effects of SAMB on VCAM-1 expression in ALI mice’s lung tissue: (**A**) Representative immunohistochemical images of VCAM-1 in lung tissue (×200). (**B**) Immunohistochemical detection of VCAM-1 expression in mouse lung tissues (n = 6). (**C**) Western blotting of VCAM-1 in mouse lung tissues. (**D**) Quantitation analysis of protein expression of VCAM-1 (n = 4). Data are shown as the mean ± SD; * *p* < 0.05 vs. normal group; # *p* < 0.05 vs. LPS+SAMB group.

The immunohistochemical analysis (Figure 5A,B) and western blotting (Figure 5C,D) demonstrated a significant reduction in VCAM-1 expression in murine lung tissues following SAMB treatment (0.5 g/kg).

## 3. Discussion

**Figure 6:**
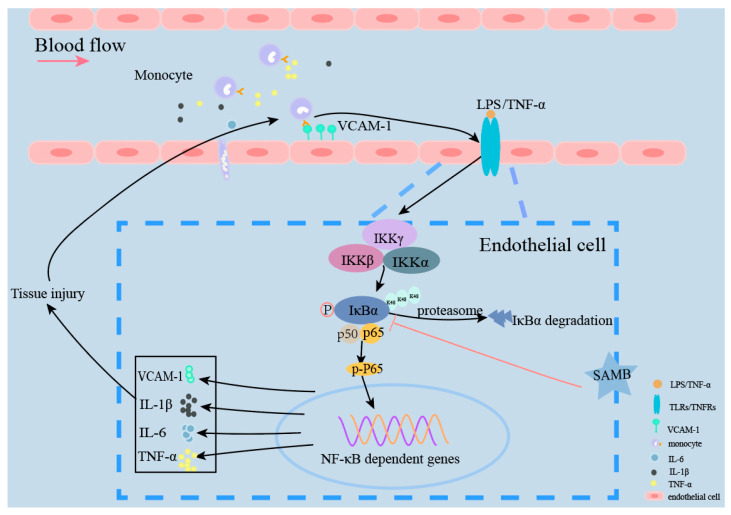
A proposed working model for the role of SAMB in regulating endothelial inflammation.

This study aimed to investigate the potential anti-inflammatory effects of SAMB on ECs both in vitro and in vivo (schematic summary shown in Figure 6). The induction of inflammation in HUVECs in vitro was achieved by employing exogenous PAMPs (LPS) or endogenous inflammatory mediators (TNF-α). The findings demonstrated that SAMB significantly reduced monocytes’ adhesion to HUVECs in both models through the downregulation of VCAM-1 expression. Further mechanistic investigations revealed that SAMB inhibited IκBα degradation, thereby suppressing the NF-κB p65 phosphorylation and NF-κB-dependent expression of pro-inflammatory cytokines, including IL-6, IL-1β, and TNF-α, as well as the adhesion molecule VCAM-1 [42]. The critical role of endothelial inflammation in ALI has been demonstrated by numerous studies [43]. Therefore, we established a murine model of septic ALI by intraperitoneal administration of LPS to substantiate SAMB’s in vivo anti-inflammatory and endothelial protection effects [40]. The results demonstrated that SAMB effectively mitigated lung injury by reducing the levels of inflammatory factors in both lung tissues and sera and attenuating the expression of VCAM-1 in lung tissue. Our findings offer valuable insights into the potential application of SAMB in treating ALI, emphasizing the imperative for clinical evaluation of its efficacy in mitigating endothelial-inflammation-related diseases.

In this study, we demonstrated that SAMB effectively suppressed the transcription and expression of inflammatory factors both locally and systemically in septic ALI. Interestingly, SAMB did not significantly reduce the mRNA levels of *Tnf-α* in lung tissues; however, notable differences were observed in serum levels. In an in vitro sepsis model using LPS-stimulated human whole blood, TNF-α transcription levels were identified within 24 h. The transcript levels of TNF-α peaked at 2–4 h and subsequently exhibited a gradual decline, while its protein levels increased and then stabilized after 4–6 h [44]. Consequently, we speculate that the peak transcription of TNF-α in blood may have already passed in LPS-stimulated mice after 24 h, but the protein level remained elevated.

Furthermore, the inhibitory effect of SAMB on the inflammation-induced increase in VCAM-1 expression has been demonstrated through both in vivo and in vitro experiments. VCAM-1 (CD106) is a 90 kDa glycoprotein that is predominantly expressed in ECs and inducible under inflammatory conditions [45]. It acts as a crucial regulator of leukocyte adhesion and transendothelial migration by interacting with α4β1 integrin, thereby conversely facilitating inflammation [46]. Importantly, VCAM-1 emerges as a promising therapeutic target in immunological disorders and inflammation. The potential therapeutic applications of the anti-VCAM-1 antibody have been extensively investigated in various conditions, including rheumatoid arthritis, asthma, transplant rejection, and cancer [13]. The transmembrane region of VCAM-1 is susceptible to proteolytic cleavage by a disintegrin and metalloproteinase, ADAM17, resulting in the generation of soluble VCAM-1 (sVCAM-1) [47]. In humans, levels of circulating sVCAM-1 are elevated in various cardiovascular diseases, including hypertension, atherosclerosis, ischemic disease, myocardial infarction (MI), stroke, heart failure with preserved ejection fraction, and atrial fibrillation [16], as well as non-insulin-dependent diabetes mellitus [48] and acute eosinophilic pneumonia [49]. VCAM-1 and sVCAM-1 have emerged as pivotal biomarkers of endothelial-inflammation-related disorders and crucial targets for clinical intervention. The inhibitory effect of SAMB on VCAM-1 expression positions it as a promising and innovative therapeutic agent for mitigating endothelial-inflammation-related disorders. The impact of SAMB on sVCAM-1 remains elusive and warrants further investigation.

The NF-κB signaling pathway exerts simultaneous regulation over the expression of inflammatory and adhesion factors in ECs [50]. Numerous pivotal regulators are present along the NF-κB pathway, including TAK1, IKK, IκBα, and RelA [42]. IκBα undergoes phosphorylation by IKK and subsequent ubiquitination by β-TrCP, resulting in proteasome-mediated degradation and activation of NF-κB [36]. The current study demonstrated that SAMB stabilized IκBα, thereby facilitating its inhibitory effects on NF-κB p65 activation induced by LPS in HUVECs. However, no significant alterations were observed in the expression and phosphorylation of IKK, implying a potential disruption in the ubiquitination or degradation of IκBα that requires further verification.

Regarding the determination of SAMB dosage, the recommended clinical dose of *Allium macrostemon* in the *Chinese Pharmacopoeia* is 10 g. The conversion coefficient for equivalent dosages between mice and humans is 9.1. Based on an adult weight of 60 kg, low, medium, and high dosages for mice are approximately 1, 2, and 4 g/kg, respectively. However, preliminary experiments conducted at these dosages revealed their excessive nature, prompting subsequent reduction to dosages of 0.5 and 1 g/kg. However, these doses of SAMB are still relatively high. Our hypothesis posits that this surplus dosage may be attributed to testing solely based on the clinical dose of *Allium macrostemon* without considering its active component, SAMB, which potentially exerts a more potent effect than *Allium macrostemon* extract, necessitating higher doses. After careful consideration, we decided to exclude the data obtained from the 1 g/kg dosage in our study. In future experiments, we intend to further decrease the SAMB concentration levels to explore a concentration range that exhibits a discernible dose–effect relationship.

The clarification of several crucial issues is still required. Firstly, the oral bioavailability of SAMB remains uncertain. Secondly, the specific active ingredients of SAMB are yet to be identified. Lastly, a comprehensive understanding of the precise mechanism underlying SAMB’s IκBα stabilization function is still lacking.

In conclusion, SAMB mitigated endothelial inflammation and ALI by inhibiting the NF-κB/VCAM-1 pathway. These findings offer novel insights into potential preventive and therapeutic strategies for various diseases associated with endothelial inflammation.

## 4. Materials and Methods

### 4.1. Materials and Chemicals

The Endothelial Cell Medium (ECM) (1001) was purchased from Sciencell. LPS (L2630) was obtained from Sigma (St. Louis, MO, USA). β-Actin antibodies (T0022) were sourced from Affbiotech (Cincinnati, OH, USA), and HSP90 antibodies (TA500494) were acquired from ORIGENE (Rockville, MD, USA). Antibodies against phospho-IKK (ab194519), IKK (ab124967), VCAM-1 (ab134074), and ICAM-1 (ab124760) were procured from Abcam (Cambridge, UK). IκBα antibodies (TP56280F) and p-IκBα antibodies (TP56280F) were purchased from Abmart (Berkeley Heights, NJ, USA). Antibodies against phospho-p65 (3033), p65 (6956s), p-JNK (4668), JNK (sc7345), p-ERK1/2 (4370), ERK1/2 (4695), p-p38 MAPK (9216s), and p38 MAPK (2371s) were purchased from Cell Signaling Technology (Danversy, MA, USA).

### 4.2. Mice

Male C57BL/6 mice, aged 7 weeks and weighing 18–22 g, were procured from Hunan Slaughter Jingda Laboratory Animal Co., Ltd., (Changsha, China), under mouse license No. SYXK (Xiang 2019-0004). Throughout the experimental period, the animals had ad libitum access to water and diet, were housed at a constant room temperature of 24 °C, and were maintained in a 12 h day/night alternating circadian environment. All animal experimental procedures were approved by the Institutional Animal Care and Use Committee of Jiangxi University of Chinese Medicine and complied with all relevant ethical regulations (JZLLSC20230254). The mice were randomly assigned to three groups: PBS, LPS (5 mg/kg), and LPS+SAMB (0.5 g/kg). Each group received pretreatment with an equal volume of either PBS or SAMB solution via gavage for one week. The lung tissues were collected 24 h after intraperitoneal injection of PBS or LPS and fixed in 4% PFA for histological analysis. Serum samples were stored at −80 °C for further analysis.

### 4.3. Cell Culture and Treatment

HUVECs were cultured with ECM. Subsequently, the cells were divided into five groups: the normal control group, the LPS or TNF-α group, and the SAMB (0.5, 1, or 2 mg/mL) groups. Before treatment with LPS or TNF-α to induce inflammation, the HUVECs were pretreated with SAMB or an equal volume of PBS for 24 h.

### 4.4. Quantitative RT-PCR

Total RNA was extracted from the lung tissue or HUVECs using the TaKaRa MiniBEST Universal RNA Extraction Kit. Then, cDNA was synthesized according to the manufacturer’s instructions. qRT-PCR was performed using the Hieff^®^ qPCR SYBR Green Master Mix. The specific primer sequences are listed in Table S1.

### 4.5. Gel Electrophoresis and Western Blotting

The proteins extracted from lung tissue or HUVECs were subjected to gel electrophoresis and Western blot analysis. The supernatant was carefully collected after centrifugation at 10,000 rpm and 4 °C for 15 min. The protein concentration was determined using BCA Protein Assay Kits (Beijing Solarbio Science & Technology, Beijing, China), following the manufacturer’s instructions. Subsequently, the samples were separated by 8% or 10% SDS-PAGE and transferred onto PVDF membranes (Millipore, Billerica, MA, USA). The membranes were blocked with a solution of 5% bovine albumin serum (BAS) for 1 h at room temperature and then incubated overnight with specific primary antibodies at 4 °C. The membranes were incubated with appropriate secondary antibodies for 1 h at room temperature on the following day. Finally, protein bands were visualized using the LI-COR Odyssey system and analyzed using ImageJ software 1.52n.

### 4.6. Wound-Healing Scratch Assay

To assess the adhesion and motility of HUVECs, a total of 2.5 × 10^5^ cells were seeded in 6-well plates. Upon reaching confluence, the cell monolayer was gently scratched using a pipette tip (1 mL) to create four distinct wound areas, followed by two rinses with PBS to eliminate non-adherent cells. Subsequently, phase-contrast light micrographs were promptly captured at timepoints immediately after scratching (0 h), as well as at 12 and 24 h intervals.

### 4.7. Cell Viability Assay

The viability of the HUVECs was assessed using a Cell Counting Kit-8 (CCK8) assay. Briefly, the HUVECs were seeded into 96-well plates at a density of 8 × 10^3^ cells per well and incubated with SAMB (0.01, 0.1, 1, 5, and 10 mg/mL) for 24 h, 48 h, and 72 h. Subsequently, each well was supplemented with 10 µL of CCK8 solution (Biosharp, Hefei, China), followed by an additional incubation period of 4 h to allow for color development. The absorbance at a wavelength of 450 nm was measured using a microplate reader (Thermo Fisher Scientific Inc., Waltham, MA, USA).

### 4.8. Cell Proliferation Assay

The HUVECs were seeded in 96-well plates at a density of 3 × 10^3^ cells per well and incubated with different concentrations of SAMB (0.5, 1, and 2 mg/mL) for 24 h, 48 h, and 72 h. Subsequently, the CCK8 reagent (20 μL) was added to each well, followed by a further incubation period of 4 h. The absorbance at a wavelength of 450 nm was measured using a microplate reader (Thermo Scientific, Waltham, MA, USA).

### 4.9. Monocyte Firm Adhesion Assay

HUVECs were seeded in 6-well plates and incubated overnight. Subsequently, they were starved with 1% FBS for 6 h. Before stimulation, the cells were pretreated with SAMB for 24 h. The cells were then stimulated with LPS (100 ng/mL) or TNF-α (10 ng/mL) for 6 h. Additionally, THP-1 cells were co-incubated with calcein-AM (Beyotime Biotechnology, Shanghai, China) for 30 min. The labeled THP-1 cells were then added to the same wells containing the HUVECs and co-incubated for 3 h. Finally, PBS was employed to wash away any non-adherent cells from the HUVECs.

### 4.10. Enzyme-Linked Immunosorbent Assay

The mice were subjected to a 24 h treatment with LPS. Blood samples were collected from the retro-orbital venous plexus and allowed to clot at 4 °C, followed by centrifugation (1500 rpm at 4 °C for 15 min). Subsequently, the sample was appropriately diluted according to the instructions provided in the kit. Finally, the levels of IL-1β, TNF-α, and IL-6 in the serum were measured.

### 4.11. Histological Assay

The mouse lungs were flushed with PBS and fixed in 4% paraformaldehyde (PFA) for 24 h. Subsequently, the fixed tissues were embedded in paraffin blocks and sectioned at a thickness of 5 μm. Hematoxylin and eosin (H&E) staining was performed to visualize pathological lung changes, while pathology scores were assigned based on previously reported criteria [37].

### 4.12. Immunohistochemistry

The mouse lungs were flushed with PBS and fixed in 4% PFA for 24 h. Subsequently, the fixed tissues were embedded in paraffin blocks and sectioned at a thickness of 5 μm. Following this, peroxidase closure solution was applied to the tissue and incubated at room temperature for 10 min. An hour-long incubation in 5% BSA was performed to prevent the non-specific binding of antibodies. The sections were then subjected to overnight incubation with primary antibodies at 4 °C. Biotin-labeled secondary antibody working solution was subsequently added and incubated at room temperature for one hour. A ten-minute incubation with HRP-labeled streptavidin followed this. Finally, the DAB Detection Kit was employed to detect VCAM-1 expression, observed under a microscope after nucleation, differentiation, blue return, and xylene transparency.

### 4.13. Statistics

All values were expressed as the mean ± SD and analyzed using GraphPad Prism (San Diego, CA, USA). Images were processed with ImageJ software. The statistical significance of differences between groups was analyzed using the 2-tailed, unpaired Student’s *t*-test, with one-way or two-way analysis of variance (ANOVA), followed by post hoc analysis. Statistical significance was determined at *p* < 0.05.

## 5. Conclusions

SAMB effectively inhibited inflammation in HUVECs induced by LPS or TNF-α, as shown by reduced expression of pro-inflammatory factors and VCAM-1 with decreased monocyte adhesion. The mechanism involves NF-κB pathway inactivation, evidenced by suppressed IκBα degradation and NF-κB p65 phosphorylation. Additionally, a murine model of LPS-induced septic acute lung injury validates SAMB’s potential therapeutic effects on endothelial inflammation and acute lung injury in vivo. These findings offer valuable insights into preventive and treatment strategies for endothelial-inflammation-related diseases.

## Data Availability

The data presented in this study are available on request from the corresponding author.

## Supplementary Materials

The following supporting information can be downloaded at: https://www.mdpi.com/article/10.3390/molecules29061239/s1.
