# Supplementary material for: Saponins from Allium macrostemon Bulbs Attenuate Endothelial Inflammation and Acute Lung Injury via the NF-κB/VCAM-1 Pathway
Source: Molecules. 2024 Mar 11;29(6):1239. doi: 10.3390/molecules29061239 (PMC10976117; doi:10.3390/molecules29061239)
Supplement: Supplementary file 1 [file molecules-29-01239-s001.zip › molecules-2873104-supplementary.docx]

**Saponins from *Allium Macrostemon Bulbs* Attenuate Endothelial Inflammation and Acute Lung Injury via the NF-κB/VCAM-1 Pathway**

Li Liu^1^, Liang Qiu^1^, Jing Xue^1^, Chao Zhong^1, 2^, Manman Qin^1^, Yifeng Zhang^1^, Chuanming Xu^1^, Yanfei Xie^1,^ *, Jun Yu^2^

^1^Center for Translational Medicine, Jiangxi University of Chinese Medicine, Nanchang, Jiangxi, China

^2^Department of Cardiovascular Sciences and Center for Metabolic Disease Research, Lewis Katz School of Medicine, Temple University, Philadelphia, PA, USA

Table S1. Primers used for RT-qPCR of gene expression.


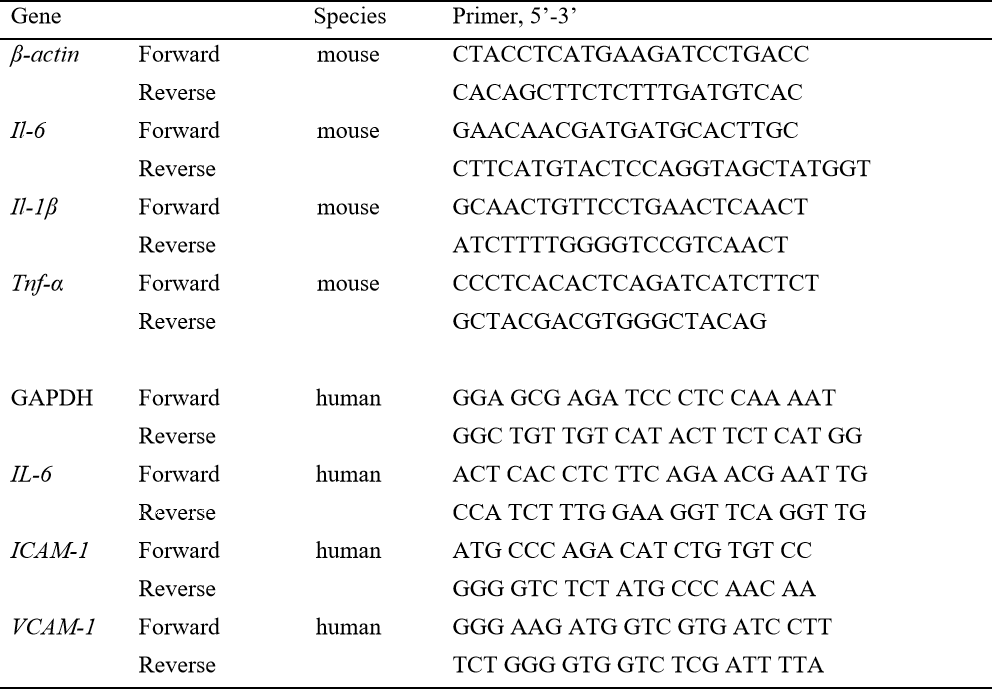


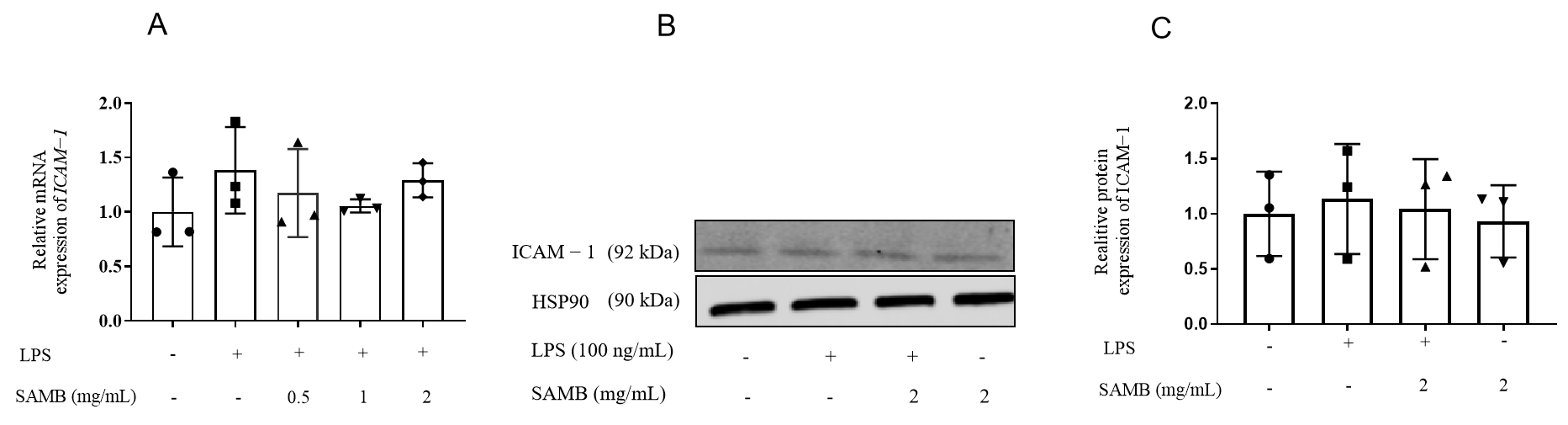


**Figure S1.** (A) The mRNA expression of *Icam-1* in HUVECs; (B) The protein expression of ICAM-1 stimulated by LPS (100 ng/mL); (C) The relative quantitation of ICAM-1 protein expression was analyzed. Data were shown as the mean ±SD. n=3.


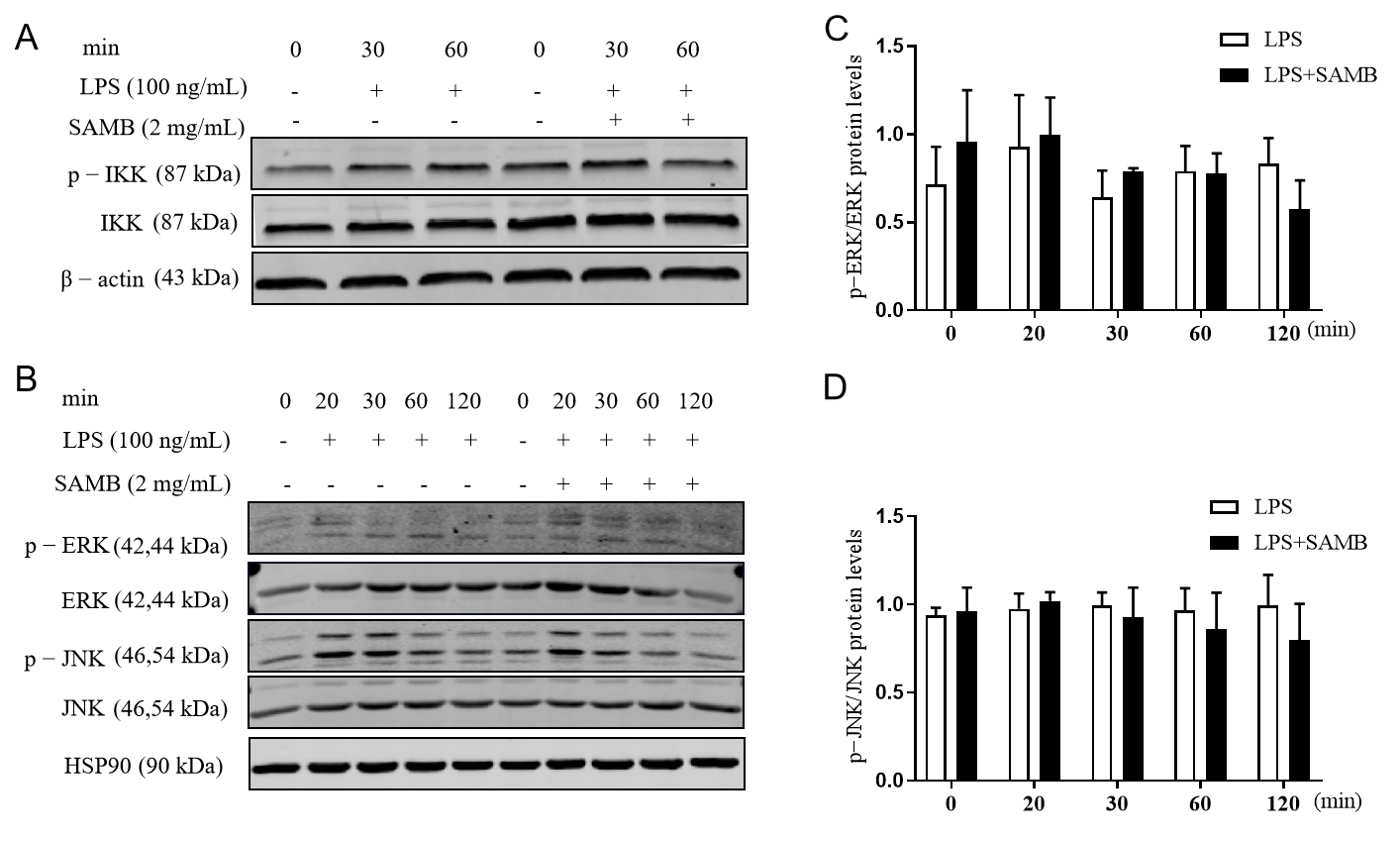


**Figure S2.** Representative images of immunoblots of p-IKK, IKK (**A**), p-ERK, ERK (**B**), p-JNK, and JNK (**B**) after LPS stimulation detected by western blotting. The relative quantitation of the p-ERK (**C**) and p-JNK (**D**) in HUVECs was analyzed. Data were shown as the mean ± SD, n = 3.
